# Mixed Neuroendocrine/Non-neuroendocrine Neoplasm (MiNEN) of the Ovary Arising from Endometriosis: Molecular Pathology Analysis in Support of a Pathogenetic Paradigm

**DOI:** 10.1007/s12022-021-09689-8

**Published:** 2021-08-03

**Authors:** Roberta Maragliano, Laura Libera, Ileana Carnevali, Valeria Pensotti, Giovanna De Vecchi, Margherita Testa, Cristina Amaglio, Eleonora Leoni, Giorgio Formenti, Fausto Sessa, Daniela Furlan, Silvia Uccella

**Affiliations:** 1 Pathology Unit, Dept. of Medicine and Surgery, University of Insubria, via O. Rossi 9, 21100 Varese, Italy; 2 Dept. of Pathology, ASST Dei Sette Laghi, Varese, Italy; 3 Cogentech Società Benefit Srl, Milan, Italy; 4 Dept. of Obstetrics and Gynecology, ASST Dei Sette Laghi, Varese, Italy

**Keywords:** MiNEN, Endometriosis, Large cell neuroendocrine carcinoma, Endometrioid carcinoma, Next-generation sequencing, Molecular pathogenesis

## Abstract

Primary ovarian neuroendocrine neoplasms (Ov-NENs) are infrequent and mainly represented by well-differentiated forms (neuroendocrine tumors — NETs — or carcinoids). Poorly differentiated neuroendocrine carcinomas (Ov-NECs) are exceedingly rare and only few cases have been reported in the literature. A subset of Ov-NECs are admixed with non-neuroendocrine carcinomas, as it occurs in other female genital organs, as well (mostly endometrium and uterine cervix), and may be assimilated to mixed neuroendocrine/non-neuroendocrine neoplasms (MiNENs) described in digestive and extra-digestive sites. Here, we present a case of large cell Ov-NEC admixed with an endometrioid carcinoma of the ovary, arising in the context of ovarian endometriosis, associated with a uterine endometrial atypical hyperplasia (EAH). We performed targeted next-generation sequencing analysis, along with a comprehensive immunohistochemical study and FISH analysis for *TP53* locus, separately on the four morphologically distinct lesions (Ov-NEC, endometrioid carcinoma, endometriosis, and EAH). The results of our study identified molecular alterations of cancer-related genes (*PIK3CA*, *CTNNB1*, *TP53*, *RB1*, *ARID1A*, and *p16*), which were present with an increasing gradient from preneoplastic lesions to malignant proliferations, both neuroendocrine and non-neuroendocrine components. In conclusion, our findings underscored that the two neoplastic components of this Ov-MiNEN share a substantially identical molecular profile and they progress from a preexisting ovarian endometriotic lesion, in a patient with a coexisting preneoplastic proliferation of the endometrium, genotypically and phenotypically related to the ovarian neoplasm. Moreover, this study supports the inclusion of MiNEN in the spectrum ovarian and, possibly, of all gynecological NENs, among which they are currently not classified.

**Supplementary Information:**

The online version contains supplementary material available at 10.1007/s12022-021-09689-8.

## Introduction

Neuroendocrine neoplasms of the ovary (Ov-NENs) are very rare and have been recently reclassified by the World Health Organization classification of tumors of female genital organs [1], on the basis of the common classification framework for NENs of different anatomical sites proposed in 2018 [2]. Morphologically, well-differentiated Ov-NENs (i.e., neuroendocrine tumors and Ov-NETs) are still called with the old term of “carcinoid” whereas poorly differentiated Ov-NENs are called neuroendocrine carcinomas (Ov-NECs) and can be of the small or large cell types [3]. The largest published series of small cells Ov-NECs reported only 11 cases [4]. Large cell NEC is even rarer and less than 50 cases have been reported in toto [3]. Both types are frequently reported in association with non-neuroendocrine carcinoma components and may be designed as ovarian mixed neuroendocrine/non-neuroendocrine neoplasms (Ov-MiNENs), in analogy to similar neoplasms arising in the digestive system and in other sites, including the urogenital tract [5, 6]. The molecular pathogenesis of Ov-NECs has been poorly addressed in the literature, to date.

Here, we report a comprehensive molecular study of a paradigmatic case of Ov-MiNEN composed of a large cell Ov-NEC and a high-grade endometrioid carcinoma, associated with ovarian atypical endometriosis and endometrial atypical hyperplasia (EAH). Each neoplastic and preneoplastic component has been separately investigated and our results support a multistep carcinogenesis and a strong genetic relationship between the neuroendocrine and non-neuroendocrine components of the Ov-MiNEN.

## Case Presentation

A 54-year-old pre-menopausal woman referred to the Gynecological Department of our Institution, in December 2018, due to abdominal discomfort and increased abdominal circumference. Abdominal computed tomography (CT) scan revealed a solid-cystic multilobed mass, with heterogeneous structure, measuring 18 × 13 × 9 cm, possibly of adnexal pertinence. Peritoneal effusion and multiple peritoneal nodules suspect for carcinosis were present. The patient underwent laparotomic hysterectomy with bilateral adnexectomy, systematic dissection of pelvic and retroperitoneal lymph nodes, with excision of all visible peritoneal nodules, omentectomy, and appendectomy. Peritoneal fluid was collected.

The macroscopic examination of the hystero-adnexectomy specimen showed a large (18 × 12 × 11 cm), solid-cystic neoplasm of the left ovary, with polycyclic margins, showing areas of necrosis and hemorrhage on the cut surface. In the uterus, the endometrial cavity showed an irregular surface on the anterior wall and multiple white nodules were observed on the serous surface, close to the left tubal corner, and were sampled and submitted. The left salpinx and the right adnexum were devoid of macroscopical lesions. The omentum showed multiple white nodules, consistent with neoplastic deposits. A total of 18 sections from the ovarian neoplasm (1 section per each cm of the largest diameter of the mass), the entire left salpinx, all the endometrial surface, and the whole right adnexum were submitted for histopathological examination. In addition, samples of the serous nodules of the uterine wall and of the omentum, as well as all peritoneal biopsies, lymph nodes, and the entire appendix, were submitted.

All surgical specimens were macroscopically observed, sampled, and routinely formalin-fixed and paraffin-embedded (FFPE). Microtomic sections obtained from paraffin blocks were stained with hematoxylin and eosin and used for immunohistochemical tests. Immunohistochemistry was performed by an automated immunostainer (BenchMark Ultra, Ventana Roche Diagnostics) and standardized protocols (Ventana OptiView DAB IHC Detection Kit), using the primary antibodies listed in Supplemental Table 1.

**Fig. 1:**
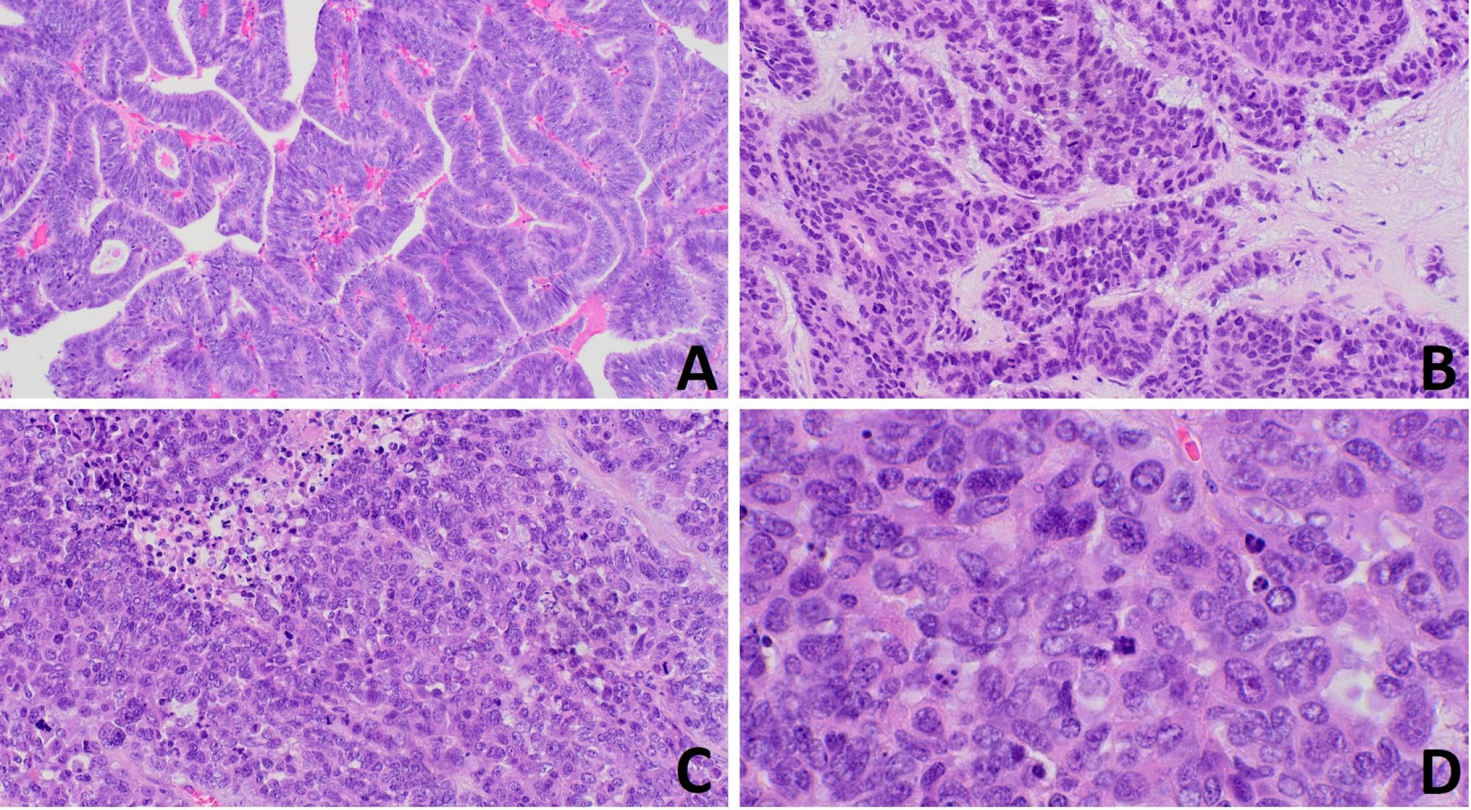
Histological features of the ovarian neoplasm. The endometrioid carcinoma component was characterized by a proliferation of back-to-back atypical endometrial-like glands (**A**), with zonal high-grade confluent growth (**B**). The large cell neuroendocrine carcinoma component showed a solid and vaguely organoid growth, with focal necrosis (**C**), and was composed of large cells with moderately abundant eosinophilic cytoplasm, round or oval nuclei with coarse chromatin, and some large eosinophilic nucleoli. Mitosis and apoptosis were common (**D**)

**Fig. 2:**
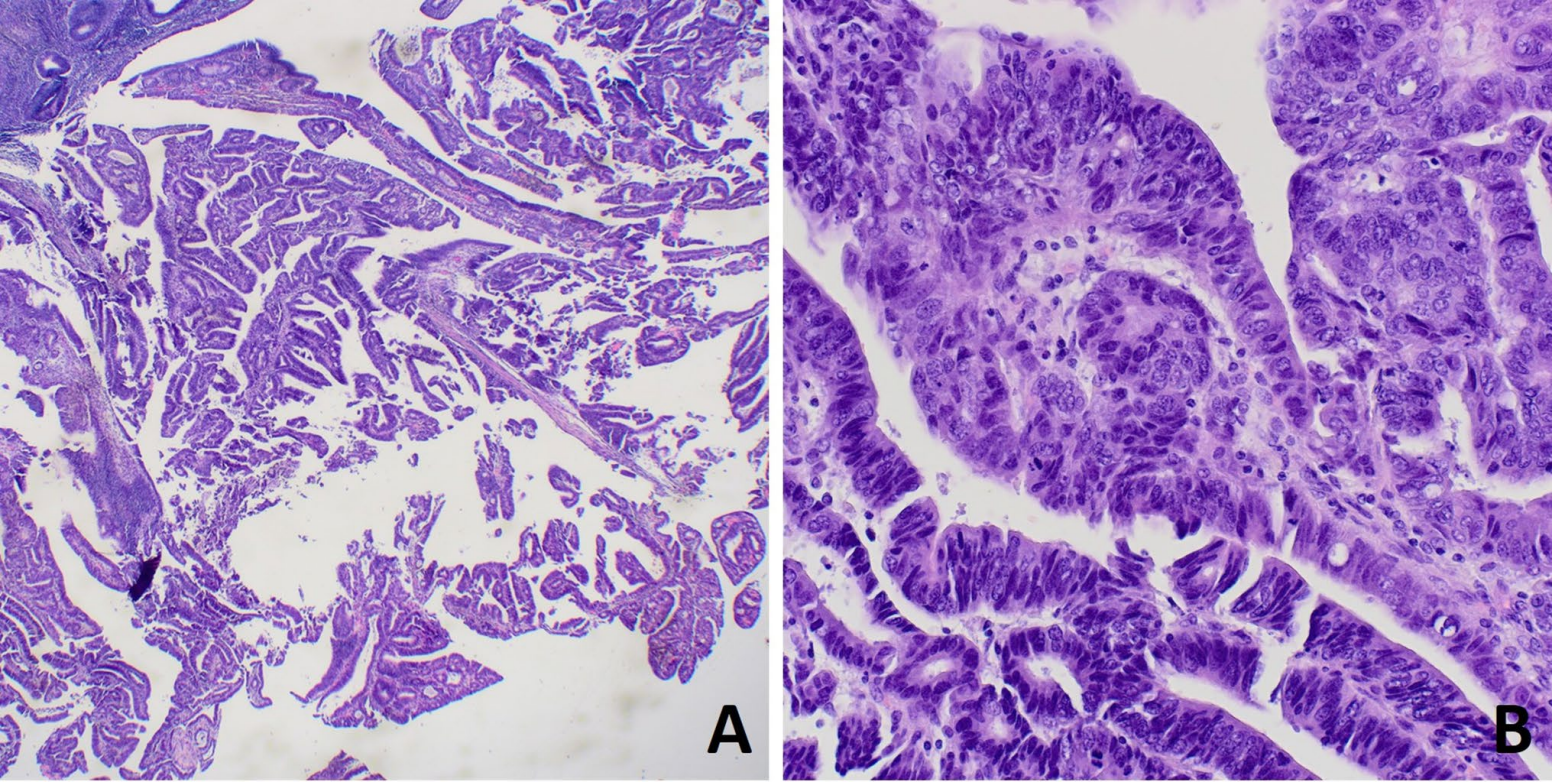
Atypical endometrial hyperplasia was seen as a polypoid lesion characterized by complex architecture with branching glands (**A**) and zonal significant nuclear atypia (**B**)

The histological slides of the left ovarian mass showed a biphasic proliferation, composed of two different neoplastic epithelial components, clearly identifiable at low power magnification, each accounting for about half of the tumor volume. The first component was characterized by a proliferation of back-to-back glandular elements lined by endometrium-like epithelium with variable degrees of atypia (Fig. 1A). Squamous differentiation was not observed. High-grade areas with more confluent growth and severe nuclear atypia were present (Fig. 1B). Adjacent and partly admixed to this carcinoma, a solid neoplastic proliferation, arranged in sheets, macronodules, and large trabeculae (Fig. 1C), was composed of epithelial cells with moderately abundant eosinophilic cytoplasm and large atypical nuclei showing coarse chromatin and evident nucleoli (Fig. 1D). In this component, mitotic index was 64 per 2 mm^2^. Interestingly, in the cystic areas of the neoplasm, foci of ovarian endometriosis were present. In the endometrium, crowding of glands, with architectural and cytological alteration, in absence of stromal invasion, consistent with atypical endometrial hyperplasia was observed (Fig. 2).

**Fig. 3:**
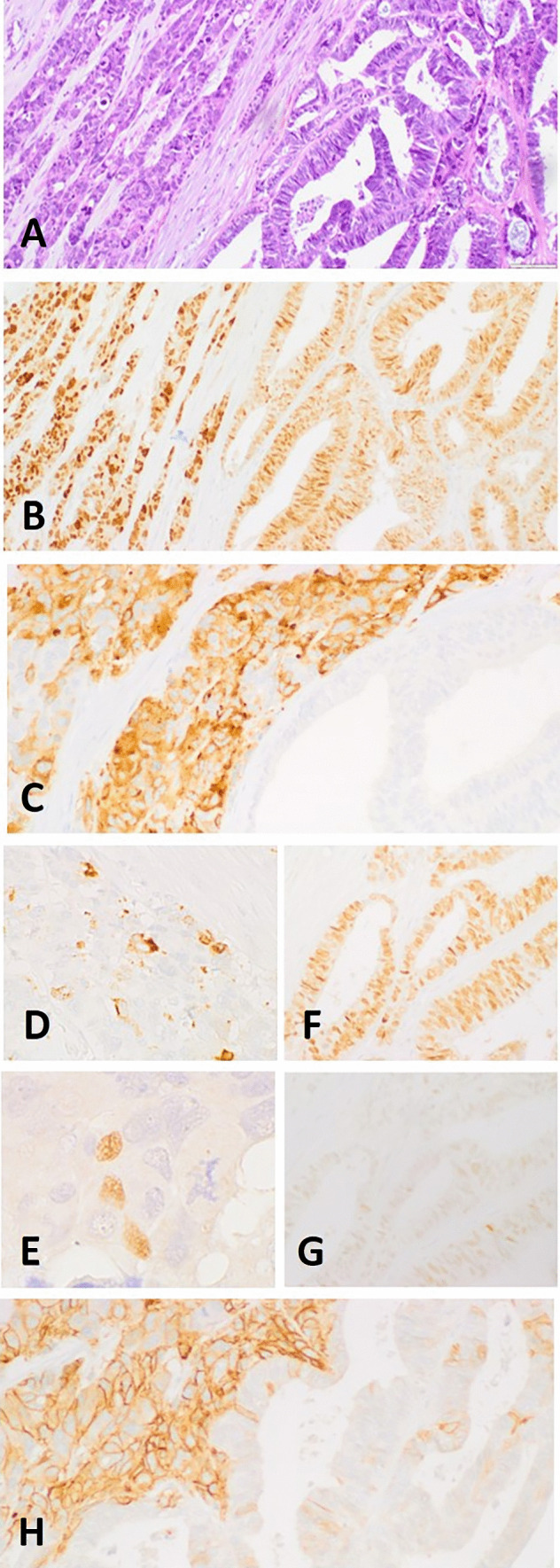
Immunohistochemical features of the Ov-MiNEN. The biphasic proliferation seen in the slides stained with hematoxylin and eosin (**A**, NEC on the left, endometrioid adenocarcinoma on the right) was evenly positive for PAX8 (**B**), whereas other markers were differentially expressed. Immunostain for synaptophysin was intensely and diffusely expressed only in the NEC component (**C**), which was also focally positive for chromogranin A (**D**) and INSM1 (**E**). The endometrioid carcinoma component was positive for ER (**F**) and, faintly, for PgR (**G**), which were negative in the NEC. SSTR2A showed membranous expression only in the NEC component (**H**)

**Fig. 4:**
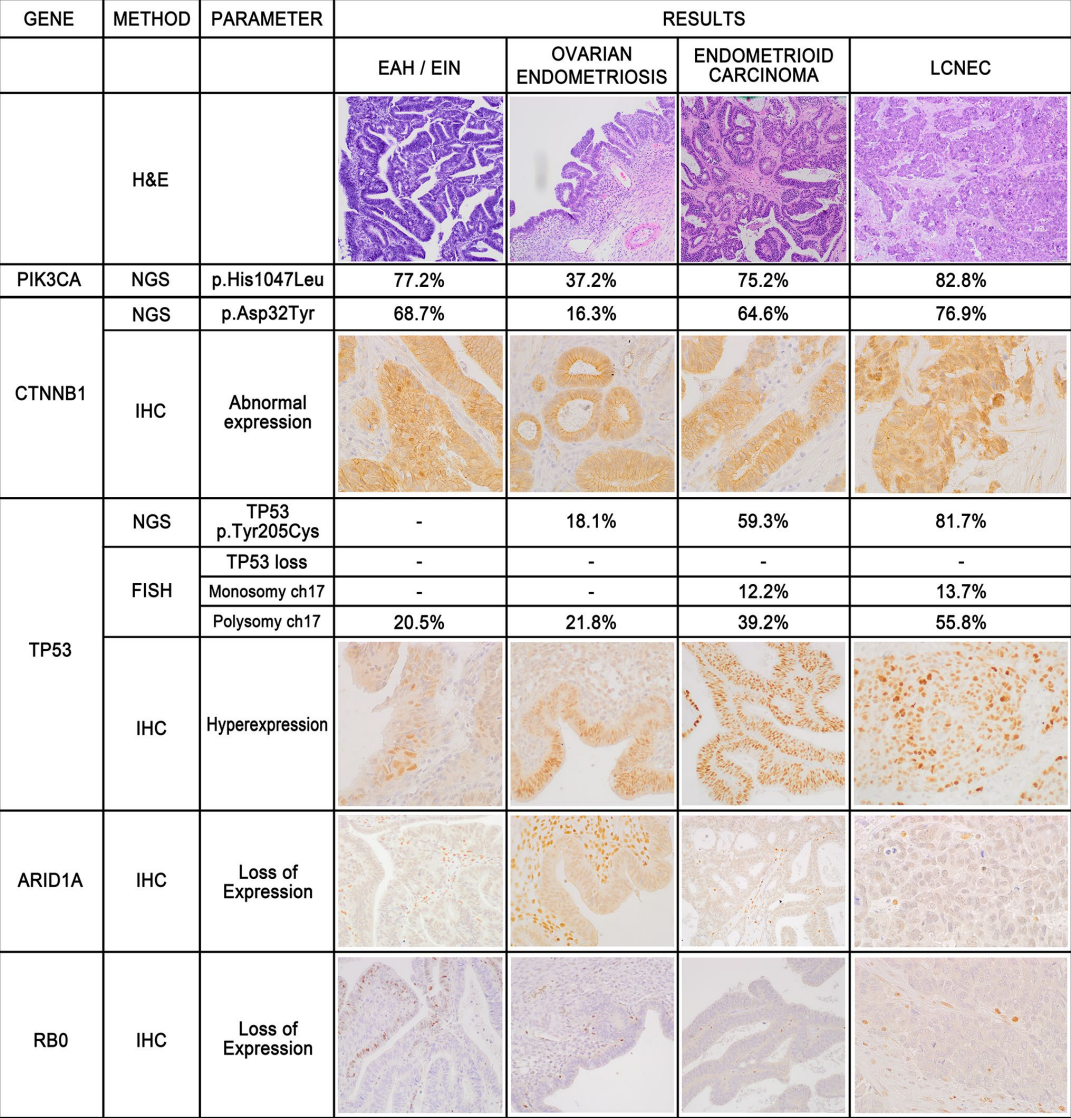
Results of the molecular and immunohistochemical study of alterations of cancer-related genes in the four morphologically distinct lesions find in the ovary and in the endometrium (see text for details)

The results of the immunohistochemical stains are illustrated in Figs. 3 and 4 and detailed in Table 1. Briefly, immunostains performed for diagnostic purposes showed PAX8 positivity and WT1 negativity in both components, whereas estrogen receptor (ER) (intense and diffuse), progesterone receptor (PgR) (intense and diffuse), and cytokeratin 7 (focal) were only expressed in the glandular component. Intense and diffuse immunoreactivity for synaptophysin and somatostatin receptor 2A (SSTR2A) and focal stain for chromogranin A, INSM1, and CD56 was only present in the solid “large cell NEC” component, which was also strongly positive for CDX2 and negative for TTF1 and cytokeratin 20. CDX2 was also focally positive in the endometrioid component. Overexpression of p53 and loss of Rb expression was observed in both components. Ki67 proliferation indices were 50% and 75% in the glandular component and solid tumor large cell carcinoma components, respectively. Additional immunohistochemical stains showed p16 overexpression with block-type stain and ARID1A loss in both components, whereas beta-catenin showed nuclear reactivity in the majority of the solid component. In turn, the glandular component showed beta-catenin nuclear reactivity in a lower proportion of cells and an abnormal pattern of expression was also focally seen in ovarian endometriosis and in uterine EAH (Fig. 4). The expression of mismatch repair (MMR) proteins (MSH2, MSH6, MLH1, and PMS2) was retained in all neoplastic cells.

**Table 1:** Immunohistochemical results for diagnostic markers in the two components of the ovarian mixed neuroendocrine/non-neuroendocrine neoplasm (Ov-MiNEN)

	LCNEC	Endometrioid carcinoma
PAX8	+ + +	+ + +
ER	-	+
PgR	-	+
WT1	-	-
Synaptophysin	+ + +	-
Chromogranin A	+	-
INSM1	+	-
CD56	+	-
CK7	-	+
CK20	+ + +	-
CDX2	+ + +	+
TTF1	+ + +	-
Ki67	70%	50%
MMR proteins	Expressed	Expressed
SSTR2A	-	3 +

Legend: *LCNEC* large cell neuroendocrine carcinoma, *ER* estrogen receptor, *PgR* progesterone receptor, *CK* cytokeratin, *INSM1* insulinoma-associated protein 1, *MMR* mismatch repair protein (MLH1, MSH2, MSH6, PMS2), *SSTR2A*, somatostatin receptor 2A (score according to Volante (14));  +  +  + : intense and diffuse expression; + : focal or weak expression

On the bases of the histopathological and immunohistochemical findings, a diagnosis of a high-grade endometrioid carcinoma (50% of the tumor volume) admixed with a large cell neuroendocrine (50% of the tumor volume) of the ovary, associated with atypical ovarian endometriosis and uterine EAH, was rendered. In analogy with similar neoplasms diagnosed in the digestive and urinary tract [5, 6], this neoplasm was designed an ovarian MiNEN. All intraabdominal metastatic deposits, including the nodules found in the omentum and on the serous surface of uterine wall and microscopic infiltration of all peritoneal biopsies and appendix, were of the NEC type. No lymph node metastasis was observed. Both salpinges and the left ovary were negative for carcinomatous infiltration. Peritoneal fluid was positive for malignant tumor cells. The neoplasm was staged pT3c, pN0, stage IIIc, according to the 8th edition of UICC/AJCC TNM Classification System.

After surgery, the patient underwent six cycles of platinum and etoposide-based adjuvant chemotherapy but experienced disease progression and she died of disease in June 2019, 6 months after initial diagnosis. As she had reported a family history with mother affected by breast cancer and oncologic genetic counselling was proposed, but the patient could not benefit of this opportunity.

## Molecular Analyses

### DNA Extraction

**Fig. 5:**
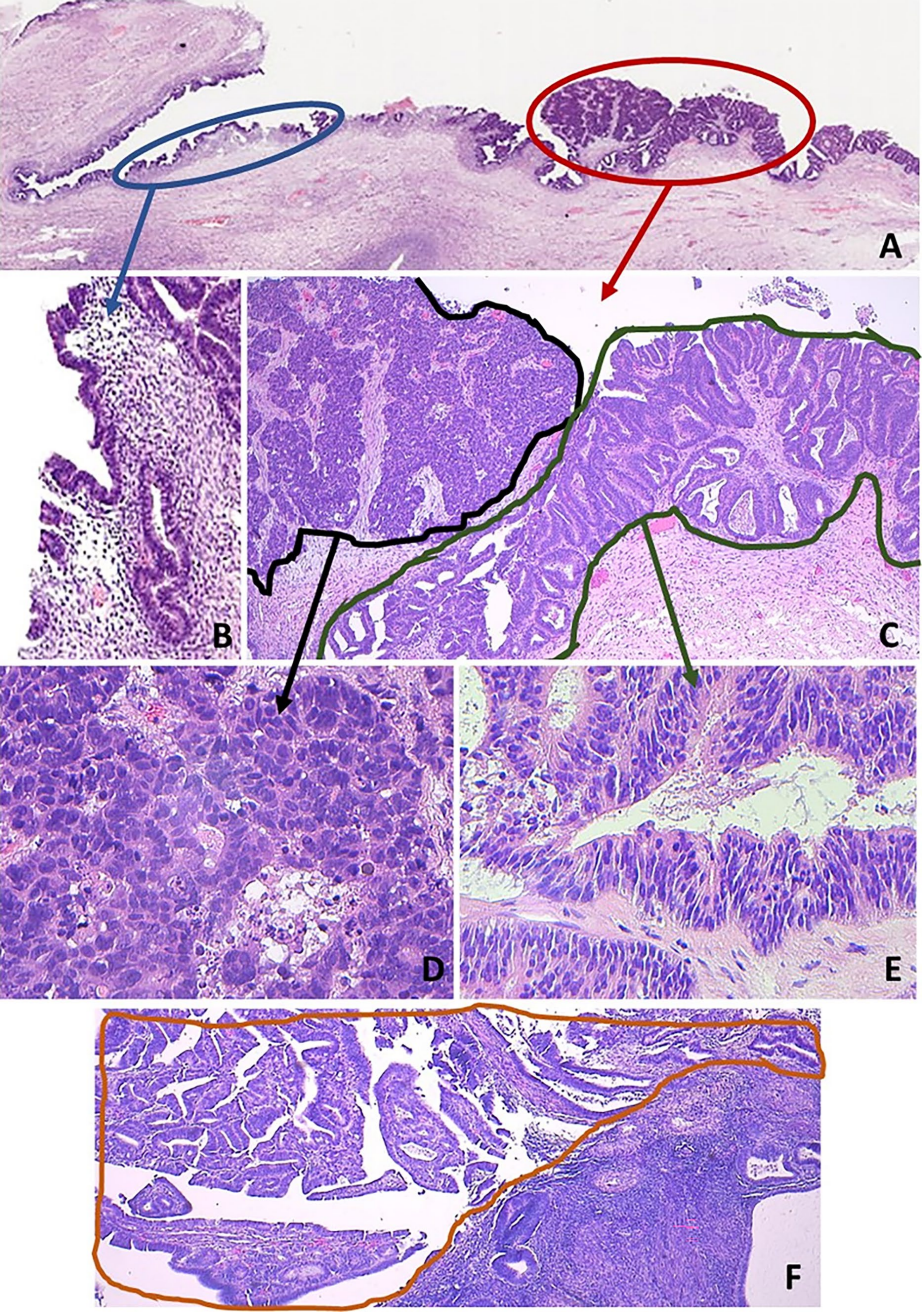
Visual representation of the criteria used for microdissection. At low power (**A**), in this section of the ovarian mass, a cystic endometriosis is visible (blue circle, enlarged in **B**), adjacent to the frankly malignant neoplasm (red circle). At higher magnification (**C**), the latter showed a biphasic morphology, composed of a solid proliferation (black line) arranged in ribbons and nests, of large cells with atypical nuclei and moderately abundant eosinophilic cytoplasm (**D**), along with a glandular endometrioid growth (green line, enlarged in **E**). In the endometrial cavity (**F**), a polypoid growth with the morphological features of the endometrial atypical hyperplasia/endometrioid intraepithelial neoplasia (orange line) was found

DNA was extracted from 8-µm-thick microtomic sections obtained from FFPE samples. In each section, diagnostic areas of ovarian NEC, endometrioid carcinoma, and endometriosis, as well as uterine EAH, were selected and manually microdissected by an expert pathologist (SU) (Fig. 5), in order to contain at least 80% of tumor cells, to minimize contamination by normal cells. DNA was extracted using Maxwell® DNA FFPE Kit and Maxwell 16 system (Promega, Madison, WI, USA) according to the manufacturer’s protocol. Each sample was quantified using Qubit dsDNA High Sensitivity Assay Kit (Invitrogen, Thermo Fisher Scientific, USA).

### Targeted Next-Generation Sequencing (NGS) Analyses

A targeted NGS analysis was performed on all the four different lesions (NEC, endometrioid carcinoma, ovarian endometriosis, and uterine EAH) and on a sample of normal tissue for comparison using the Human Actionable Solid Tumor Mutations QIAseq DNA Panel (DHS-101Z, Qiagen, Hilden, Germany) that analyses 22 oncogenes (*BRAF*, *PDGFRA*, *EGFR*, *KRAS*, *NRAS*, *KIT*, *AKT1*, *ALK*, *CTNNB1*, *ERBB3*, *ESR1*, *FOXL2*, *GNA11*, *GNAQ*, *IDH1*, *IDH2*, *MET*, *RAF1*, *RET*, *ERBB2*, *PIK3CA*, and *TP53*). A targeted amplicon-based library was constructed as described in a previous work of our group [7] according to the manufacturer’s protocol. Barcoded libraries were quantified, pooled together at 8 pM, and sequenced on an Ion S5 XL System (A27214, Thermo Fisher Scientific) using Ion 530 Chip (Thermo Fisher Scientific) as previously reported [7]. Unmapped BAM (uBAM) files were imported into the CLC Genomics Workbench (Qiagen Bioinformatics, Germany, version 12) and mapped on the Human hg19 genome. Sequencing data were analyzed as previously reported [7] and they were filtered ensuring a coverage of at least 100 × and a variant allele frequency (VAF) of 5%.

Since the patient had a family history suggestive for a hereditary cancer syndrome with mother affected by breast cancer, a second NGS analysis addressing 37 genes involved in hereditary cancer syndromes was performed by Cogentech Laboratory (Milan, Italy) using a custom panel, named OncoPan®, that can detect single nucleotide variants (SNV), small ins/del, and CNV of the following genes: *APC*, *ATM*, *BARD1*, *BMPR1A*, *BRCA1*, *BRCA2*, *BRIP*, *CDH1*, *CDKN2A* (α e β), *CDK4* (exon 2), *CHEK2*, *CTNNA1*, *EPCAM*, *FANCM*, *MLH1*, *MSH2*, *MSH3*, *MSH6*, *MUTYH*, *NBN*, *NHTL1*, *PALB2*, *PMS2*, *POLD1*, *POLE*, *PTEN*, *RAD51C*, *RAD51D*, *SMAD4*, *STK11*, *TP53*, *KRAS*, *NRAS*, *BRAF*, *EGFR*, *HER2 (ERBB2)*, and *PIK3CA*. About 150–200 ng of dsDNA, according to Qubit dsDNA HS assay kits fluorimetric quantification, were sheared by Sure Select Enzymatic Fragmentation Kit (Agilent Technologies Inc.). NGS library was created using Sure Select XT2 Low Input Custom Library Probes (Agilent Technologies Inc.), and sequencing was performed on MiSeq (Illumina Inc., San Diego, USA) using 2 × 150 bp paired-end sequencing. Data collection was performed with the MiSeq Reporter (MSR) software v.2.6.2.3, using the “FastQ only” workflow. The run quality was evaluated by Illumina Sequencing Analysis Viewer v.1.9.1, while bioinformatics pipeline for the annotation of the vcf files and CNV calls was developed in-house, in collaboration with enGenome Software Company (Pavia, Italy). Paired-end reads were mapped on the Human hg19 genome. The variants identified were filtered with a minimum coverage of 50 × and a VAF of at least 10%. Finally, the pathogenic variants of *TP53* and *PIK3CA* identified were confirmed with Sanger sequencing. The regions were amplified at the annealing temperature of 60 °C, with AmpliTaq Gold Kit (Applied Biosystems; Thermo Fisher Scientific, Inc., Waltham, MA, USA). Sequencing was performed on purified PCR products by using BigDye® Terminator v.3.1 Cycle Sequencing Kit (Thermo Fisher Scientific, Inc.) and run 3500 Dx Genetic Analyzer (Life Technologies). Sequences were analyzed by Mutation Surveyor® Software (v5.1.0; SoftGenetics, LLC., State College, PA, USA). Primers’ sequences used are available under request.

### Fluorescent In Situ Hybridization (FISH) Analysis

Interphase FISH analysis was performed on 3–4-μm sections used for conventional histological examination, according to the guidelines of the European Cytogeneticists Association (European Cytogenetic Guidelines: www.e-c-a.eu). The experiments were carried out as previously described [8]. The following probes were hybridized: TP53/Cen17 Dual Color Probe (Zytovision GMBH, Germany) that simultaneously hybridizes the TP53 gene (red labeled) and centromere of chromosome 17 (green labeled); Pathvision (Vysis) probe that simultaneously hybridizes Her2/neu gene (red labeled), and the centromere of chromosome 17 (green labeled) and *CDK4*/CEN 12 (Zytovision GMBH, Germany) that simultaneously hybridizes *CDK4* gene (green labeled) and the centromere of chromosome 12 (red labeled). FISH analysis was performed using a BioView (Abbott) and tissue matching procedure. Only experiments with 90% hybridization efficiency were considered. *TP53*, *HER2*, and *CDK4* loss was considered when in each cell, specific gene signals were fewer than reference signals. Monosomy (one specific gene and one reference centromeric signal) was also counted as loss of specific gene. Polysomies were evaluated when more than two centromeres were counted and amplification gene was evaluated when ratio between gene signals and centromeres was > 2. The cut-off value was 10% for both monosomy and polysomy.

### Molecular Results

NGS analysis with Human Actionable Solid Tumor Mutations QIAseq DNA Panel performed on all the four patient’s specimens showed a good coverage with a mean read depth of 977 × (363 × minimum coverage and 1512 × maximum coverage). Before any filter was applied, 36 variants were identified for NEC, 33 variants for endometrioid carcinoma, 34 for endometriosis, and 192 for uterine EAH. These variants were filtered with a coverage of at least 200 × and a VAF of 5%. In order to detect only variants with a deleterious effect on protein functions, both synonymous and 1000 Genome Project variants were filtered out and the resulting variants are reported in Fig. 3. All the four specimens exhibit the same percentage of neoplastic cell content (80–90%); thus, they resulted absolutely comparable to each other. In all the four components, two pathogenic mutations, known to be recurrent hotspots in cancer, were present: *PIK3CA* c.3140A > T p.(His 1047Leu) and *CTNNB1* c.94G > T p.(Asp32Tyr). *PIK3CA* variant was identified with VAF 82.8% in NEC, 75.2% in endometrioid carcinoma, 37.2% in ovarian endometriosis, and 77.2% in uterine EAH, while *CTNNB1* variant was identified with VAF 76.9% in NEC, 64.6% in endometrioid carcinoma, 16.3% in ovarian endometriosis, and 68.7% in uterine EAH (Fig. 3). Moreover, the targeted NGS analysis identified the *TP53* gene missense mutation c.614A > G p.(Tyr205Cys) in NEC (VAF 81.7%), endometrioid carcinoma (VAF 59.3%), and ovarian endometriosis (VAF 18.1%), not detected in EAH of the endometrium (Fig. 3). This missense variant replaces tyrosine with cysteine at codon 205 of *TP53* (p.Tyr205Cys). Numerous experimental studies in model organisms have shown that this missense change disrupts the transcriptional transactivation activity of TP53 [9, 10]. Although high VAFs were observed in the pathological samples, *PIK3CA*, *CTNNB1*, and *TP53* gene variants were absent in the normal tissue, excluding any possible germline mutation in these genes.

NGS analysis with OncoPan® Panel of NEC and endometrioid carcinoma showed a full coverage of the coding regions, 270 × mean read depth and read coverage of > 50 × was obtained (200 × minimum coverage and 1435 × maximum coverage on average). No pathogenic variants in genes involved in *BRCA1/BRCA2*, Lynch, or Cowden syndromes were identified. As expected, this second NGS panel confirmed the presence of *TP53* c.614A > G p.(Tyr205Cys) and *PIK3CA* c.3140A > T p.(His 1047Leu) in both samples with VAF comparable to the first NGS analysis. Finally, this NGS approach revealed gains of different regions including *MUTYH*, *CDK4*, *KRAS*, *POLE*, *ERBB2*, *TP53*, *POLD1*, and *STK11* in both NEC and endometrioid tumors.

#### FISH Analysis

FISH analysis with *TP53* probe was performed on complex atypical endometrial hyperplasia, ovarian endometriosis, and on both exocrine and endocrine components of ovarian carcinoma. No loss of *TP53* signal was observed in each sample. However, monosomy of chromosome 17 suggestive for loss of *TP53* was observed in 13.7% and 12.2% of hyperplasia and endometriosis cells, respectively. In contrast, monosomy of chromosome 17 was below the cut-off value in both exocrine and endocrine components of the ovarian carcinoma. Interestingly, a polysomic cell population for chromosome 17 was observed in all samples, and it was the more represented cell population in the malignant components, compared to hyperplasia and endometriosis (Fig. 3). *HER2* and *CDK4* regions were analyzed in both adenocarcinoma and neuroendocrine carcinoma components of Ov-MiNEN and neither HER2 nor CDK4 amplification was observed, albeit polysomies of chromosomes 12 and 17 were found.

## Discussion

Primary neuroendocrine carcinoma of the ovary (Ov-NEC) is a very rare entity, the pathogenesis of which is still far from being elucidated. A significant proportion of Ov-NECs, particularly of the large cell type, is reported to be associated with non-neuroendocrine components [3], suggesting an histogenetic and pathogenetic relationship with other ovarian neoplasms, mostly including surface epithelial tumors. Thus, the inclusion of the concept of MiNEN in the spectrum of gynecological NENs, in analogy with digestive and extra-digestive locations [5, 6], may be proposed. However, the existence of common driving genetic alterations in the neuroendocrine and non-neuroendocrine components of Ov-MiNENs has been poorly addressed in the literature.

The case we have reported here is composed by two morphologically distinct and well recognizable neoplastic population: a large cell neuroendocrine carcinoma (LCNEC) and a high-grade endometrioid carcinoma of the ovary. The immunohistochemical study confirmed the biphasic phenotype of the proliferation, by demonstrating the expression of general neuroendocrine markers and SSTR2A limited to LCNEC, which, in turn, was negative for hormonal receptors (estrogen and progesterone receptors). Interestingly, a strong and diffuse immunostain for the transcription factor PAX8, which is a marker of Mullerian epithelia, was present in both histotypes, as an early clue to the common ontogenesis of the two components. However, the LCNEC and, to a lesser extent, the endometrioid carcinoma also expressed CDX2, a homeobox protein expressed in intestinal epithelial cells and related proliferation. This confirms, on one hand, the aleatory significance of transcription factors in the diagnostic management of NECs [11] and, on the other hand, the aberrant expression of CDX2 in endometrioid carcinoma, which has been well documented, especially in tumor with *CTNNB1* mutation and with squamous differentiation, but has also been demonstrated in absence of squamous morulae [12]. Further immunohistochemical analysis, oriented to define the molecular profile of the two components, showed substantial identity of the two components (loss of RB protein expression, diffuse overexpression of p53, block-type p16 overexpression, loss of ARID1A, and altered beta-catenin expression). Interestingly, both ovarian endometriosis and AEH/EIN shared the same altered immunohistochemical profile of the frankly neoplastic components.

The molecular study, performed separately on the two components of the Ov-MiNEN, substantially supported the existence of a common pathogenetic pathway for both LCNEC and endometrioid carcinoma and was largely in agreement with immunohistochemical results. LCNEC and endometrioid carcinoma showed the same mutational and CNVs profiles. In details, NGS detected three pathogenetic mutations in both components, respectively in *TP53*, *PIK3CA*, and *CTNNB1* genes, with similar variant allele frequency (VAF). The mutations of *PIK3CA* and *CTNNB1* genes, as well as the alteration of the SWI/SNF complex, that was documented in our case by the loss of ARID1A expression, are among the most common and earliest genetic abnormalities detected in endometrioid carcinoma [1]. In turn, *TP53* mutations and also its altered immunohistochemical patterns are associated with a worse prognosis in these neoplasms [13]. The coexistence, in the LCNEC component, of the same genetic alterations observed in the endometrioid carcinoma rules out the possibility of a collision between an ovarian endometrioid carcinoma and a metastatic LCNEC from another primary site. Furthermore, it strongly supports the hypothesis that NECs are, in fact, genetically related to non-neuroendocrine carcinomas of a given anatomical site, as already demonstrated in the large intestine and in the pancreas and suggested in other sites, such as the urinary bladder [14]. Indeed, in the gynecological tract, the molecular subtyping of endometrial carcinoma has also been applied to endometrial NECs, giving further consistence to this view and shading light into new scenarios for the clinical management of these neoplasms [15]. In our case, the high similarity shared by the two components is not in favor of a step-wise progression from the endometrioid to the neuroendocrine component through the accumulation of additional genetic defects, as it has been proposed in other sites [14]. Nevertheless, the greater biological aggressiveness of the LCNEC component, compared to the non-neuroendocrine one, is witnessed by the fact that all metastatic deposits were represented by LCNEC only. We can, thus, speculate that additional genetic or epigenetic alterations may be implicated in the acquisition of the neuroendocrine phenotype and of the greater propension to dissemination of neoplastic cells. In fact, the biologic aggressivity and the peculiar therapeutic approach of NECs make their proper recognition and distinction from undifferentiated/dedifferentiated non-neuroendocrine carcinomas an important challenge for pathologists. The two entities have been thought to largely overlap for a long time, especially for what LCNEC is concerned, but the definition of clear-cut criteria for the diagnosis of this latter entity gives important support for correctly identifying it. A distinctive neuroendocrine morphology is requested, including organoid growth, low nuclear-cytoplasmic ratio, vesicular nuclei with evident nucleoli, eosinophilic, or amphophilic, frequently granular, cytoplasm. In presence of such morphology, the immunoreactivity for general neuroendocrine markers (preferentially chromogranin A, and when chromogranin A is negative, diffuse positivity for synaptophysin and INSM1) is diagnostic for LCNEC. Positivity alone for synaptophysin or non-specific markers (e.g., CD56, CD57, and NSE) should be used to make the diagnosis of LCNEC, even when dealing with a indefinitely poorly differentiated morphology [15, 16].

One of the most intriguing aspects of our case was the association of this neoplasm with foci of ovarian endometriosis and with atypical endometrial hyperplasia (AEH). It is well-known that most of endometrioid and clear cell carcinomas of the ovary arise in association with ovarian endometriosis [17]. This epidemiological observation has been recently supported by the molecular demonstration of the presence of cancer-associated mutations in non-neoplastic endometriosis, which seem to be boosted in the ovarian microenvironment (and not in other pelvic sites), where neoplastic transformation occurs [18–22]. Nevertheless, a direct link of endometriosis with ovarian neuroendocrine carcinoma has never been reported. In the case described here, a clear molecular relationship between the endometriotic lesion and both Ov-MiNEN components emerges when immunohistochemical and genetic results are compared with morphology. The driver mutations in cancer-related genes (namely *PIK3CA* and *CTNNB1*) identified in the malignant proliferation are also recognizable, even if with a lower VAF, in the endometriotic lesion, supporting the progression of the neoplastic clone from endometriosis cells that, in turn, showed morphological signs of dysplastic transformation. Interestingly, a low frequency of TP53 mutation was also found in the endometriotic tissue. This finding, previously reported by another study [23], further supports the preneoplastic nature of ovarian endometriosis. The immunohistochemical analysis confirmed and strengthened these findings, as p53 overexpression, altered beta-catenin expression pattern, and loss of ARID1A expression were observed in endometriosis, as well. Furthermore, the same mutation in *PIK3CA* and *CTNNB1* genes found in the ovarian proliferations was also observed in AEH, which showed loss of ARID1A expression and some extent of beta-catenin nuclear translocation. In addition, the immunohistochemical overexpression of p53 in focal areas of AEH, together with a certain degree of monosomy of *TP53* locus, is in support of an initial abnormality (possible first hit) of this gene even in the endometrial lesion. This molecular landscape of AEH in this patient makes us speculate on the possible relationships between the endometrial and the ovarian proliferations. Finally, the unusual focal loss of Rb expression in the endometrial lesion, in view of the more widespread loss in the Ov-MiNEN, further strengthens such possibility. In turn, the ovarian neoplasm cannot be regarded to as a metachronous localization of the endometrial proliferation because no sign of stromal infiltration was found, despite the endometrium was fully submitted for histopathological examination. A putative mechanism that links the endometrial and the ovarian lesions in these patients may be found in the retrograde flow of endometrial cells already harboring cancer-associated mutations, with selective growth advantages leading to the development of endometriosis [21] that, in the ovarian microenvironment, finds a hormonal and inflammatory background favoring its neoplastic transformation [22].

## Conclusion

**Fig. 6:**
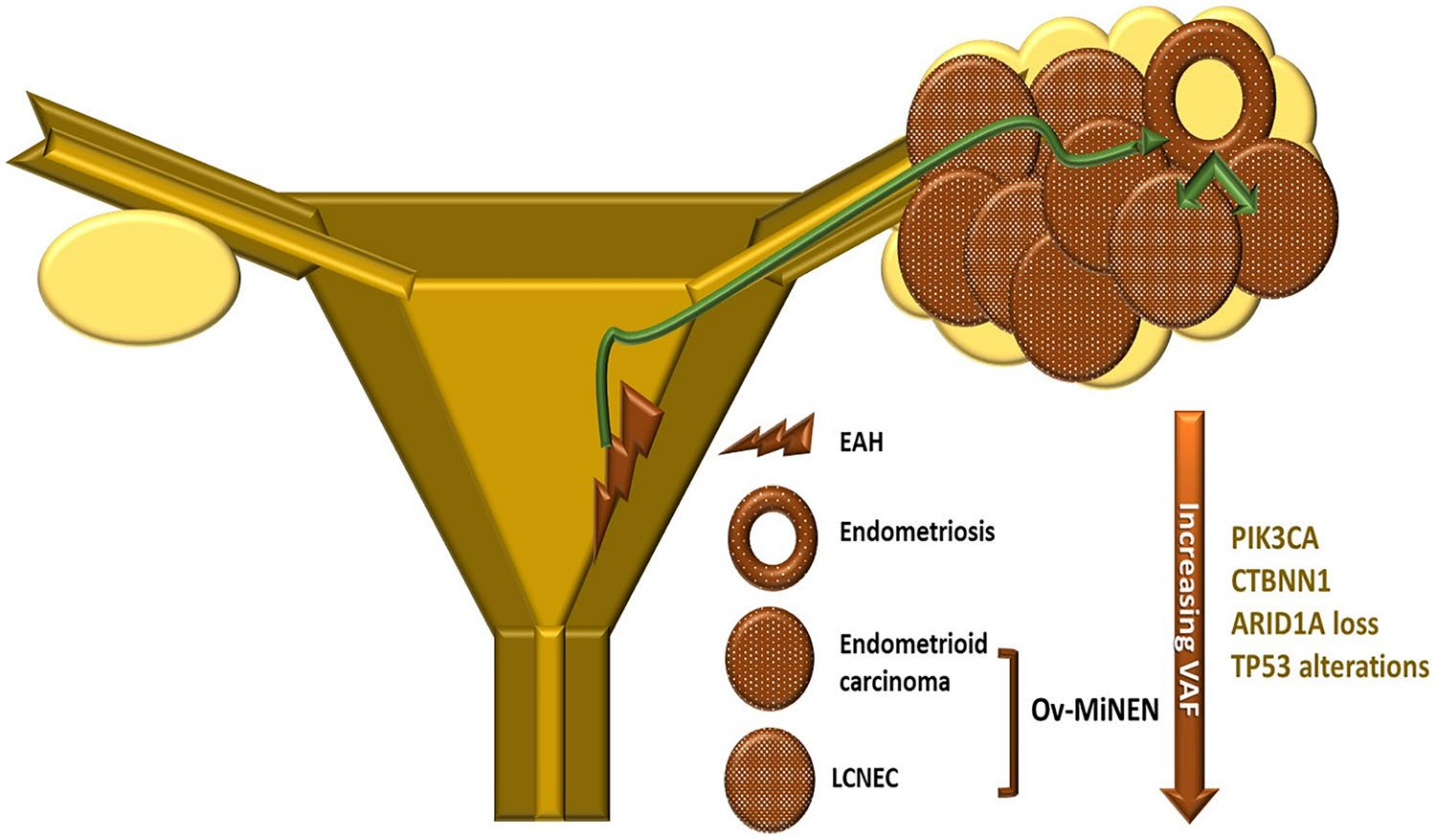
Schematic representation of the proposed pathogenetic model for the carcinogenesis of ovarian MiNEN

We described a rare case of mixed ovarian carcinoma composed of a high-grade endometrioid carcinoma and a LCNEC, which is entitled to be defined Ov-MiNEN. We demonstrated the substantial identity of the molecular profile of the two neoplastic components and their progression from a preexisting ovarian endometriotic lesion, in a patient with a coexisting preneoplastic proliferation of the endometrium, genotypically and phenotypically related to the ovarian neoplasm (Fig. 6). This case represents a paradigm for the pathogenesis of high-grade neuroendocrine neoplasia in the ovary and supports the concept that NECs arise along the same pathogenetic pathways of autochthonous non-neuroendocrine carcinomas of each specific anatomical site. Moreover, this study supports the inclusion of MiNEN in the spectrum ovarian and, possibly, of all gynecological NENs, among which they are currently not classified [1].

## Supplementary Information

Below is the link to the electronic supplementary material.

Supplementary file1 (DOCX 22 KB)
