# Supplementary material for: Mixed Neuroendocrine/Non-neuroendocrine Neoplasm (MiNEN) of the Ovary Arising from Endometriosis: Molecular Pathology Analysis in Support of a Pathogenetic Paradigm
Source: Endocr Pathol. 2021 Aug 3;33(3):400–10. doi: 10.1007/s12022-021-09689-8 (PMC9420090; doi:10.1007/s12022-021-09689-8)
Supplement: Supplementary file 1 — Supplementary file1 (DOCX 22 KB) [file 12022_2021_9689_MOESM1_ESM.docx]

**Table 1.** Antibodies used for immunohistochemical analysis.

| **Primary Antibody** | **Clone** | **Working Solution** | **Manufacturer** |
| --- | --- | --- | --- |
| ARID-1A (rabbit polyclonal) | polyclonal | 1:400 | Sigma |
| β-catenin (mouse monoclonal) | (E-5): sc-7963 | 1:500 | Santa Cruz |
| CD56 (mouse monoclonal) | 123C3 | 1:1 | Ventana |
| CDX2 (rabbit monoclonal) | EPR2764Y | 1:1 | Ventana |
| ChrA (mouse monoclonal) | LK2H10 | 1:2 | Ventana |
| CINtech P16 histology (mouse monoclonal) | E6H4 | 1:2 | Ventana |
| CK7 (rabbit monoclonal) | SP52 | 1:2 | Ventana |
| CK19 (mouse monoclonal) | A53-B/A2,26 | 1:2 | Ventana |
| CK20 (rabbit monoclonal) | SP33 | 1:1 | Ventana |
| ER (rabbit monoclonal) | SP1 | 1:1 | Ventana |
| INSM1 (mouse monoclonal) | (A-8): sc-271408 | 1:200 | Santa Cruz |
| Ki-67 (mouse monoclonal) | MIB-1 | 1:100 | Dako |
| MLH1 (mouse monoclonal) | M1 | 1:1 | Ventana |
| MSH2 (mouse monoclonal) | G219-1129 | 1:1 | Ventana |
| MSH6 (rabbit monoclonal) | SP93 | 1:1 | Ventana |
| p53 (mouse monoclonal) | DO-7 | 1:500 | Dako |
| PAX8 (mouse monoclonal) | MRQ-50 | 1:1 | Ventana |
| PgR (rabbit monoclonal) | 1E2 | 1:1 | Ventana |
| PMS2 (mouse monoclonal) | A16-4 | 1:1 | Ventana |
| Rb (mouse monoclonal) | G3-245 | 1:100 | BD Pharmingen |
| SSTR2A (rabbit monoclonal) | UMB1 | 1:400 | Abcam |
| Syn (rabbit monoclonal) | SP11 | 1:1 | Ventana |
| TTF-1 (rabbit monoclonal) | SP141 | 1:1 | Ventana |
| WT1 (mouse monoclonal) | 6F-H2 | 1:1 | Cell-Marque |

**Legend:** ARID1a – AT-rich interaction domain 1A; ChrA – Chromogranin A; CK – cytokeratin; ER – estrogen receptor; INSM1 – Insulinoma-associated protein 1; MLH1 – mutL homolog 1; MSH2 – mutS homolog 2; MSH6 – mutS homolog 6; PgR – progesterone receptor; PMS2 – PMS1 homolog 2; Rb –Retinoblastoma protein; SSTR2A – Somatostatin receptor 2A; Syn – synaptophysin; TTF-1 – transcriptional thyroid factor 1; WT1 – Wilms’ tumor protein.
